# Pulmonary Embolism in Hospitalized COVID-19 Patients in Romania: Prevalence, Risk Factors, Outcomes

**DOI:** 10.3390/v17101342

**Published:** 2025-10-05

**Authors:** Diana-Maria Mateescu, Adrian-Cosmin Ilie, Ioana Cotet, Cristina Guse, Camelia-Oana Muresan, Ana-Maria Pah, Marius Badalica-Petrescu, Stela Iurciuc, Maria-Laura Craciun, Adina Avram, Alexandra Enache

**Affiliations:** 1 Department of General Medicine, Doctoral School, “Victor Babes” University of Medicine and Pharmacy, Eftimie Murgu Square 2, 300041 Timisoara, Romania; diana.mateescu@umft.ro (D.-M.M.); ioana.cotet@umft.ro (I.C.); cristina.marin@umft.ro (C.G.); 2 Department of Public Health and Sanitary Management, “Victor Babes” University of Medicine and Pharmacy, Eftimie Murgu Square 2, 300041 Timisoara, Romania; 3 Legal Medicine, Timisoara Institute of Legal Medicine, 300041 Timisoara, Romania; muresan.camelia@umft.ro (C.-O.M.); enache.alexandra@umft.ro (A.E.); 4 Ethics and Human Identification Research Center, “Victor Babes” University of Medicine and Pharmacy, Eftimie Murgu Square 2, 300041 Timisoara, Romania; 5 Discipline of Forensic Medicine, Bioethics, Deontology, and Medical Law, Department of Neuroscience, “Victor Babes” University of Medicine and Pharmacy, Eftimie Murgu Square 2, 300041 Timisoara, Romania; 6 Cardiology Department, “Victor Babes” University of Medicine and Pharmacy, Eftimie Murgu Square 2, 300041 Timisoara, Romania; anamaria.pah@umft.ro (A.-M.P.); marius.badalica-petrescu@umft.ro (M.B.-P.); laura.craciun@umft.ro (M.-L.C.); 7 Department of Internal Medicine I, “Victor Babes” University of Medicine and Pharmacy, Eftimie Murgu Square 2, 300041 Timisoara, Romania; avram.adina@umft.ro

**Keywords:** anticoagulation, COVID-19, d-dimer, Eastern Europe, fibrinogen, pulmonary embolism, thrombosis

## Abstract

(1) Background: Pulmonary embolism (PE) is a severe complication of coronavirus disease 2019 (COVID-19), particularly in hospitalized patients. Data from Eastern Europe, including Romania, are limited, despite potential regional differences in demographics, comorbidities, and thromboprophylaxis practices. (2) Methods: This retrospective cohort study included 395 adults hospitalized with RT-PCR-confirmed COVID-19 at the “Victor Babeș” Clinical Hospital of Infectious Diseases and Pneumophthisiology, Timișoara, Romania, from September 2022 to December 2024. Demographic, clinical, laboratory, and imaging data were extracted from medical records. PE was confirmed by computed tomography pulmonary angiography (CTPA). Group comparisons used chi-square and *t*-tests, with multivariable logistic regression to identify independent PE predictors. (3) Results: PE was diagnosed in 47 patients (11.9%). Compared to those without PE, patients with PE had higher D-dimer (5305.00 ± 1251.00 vs. 537.00 ± 203.00 ng/mL, *p* < 0.001), fibrinogen (6.33 ± 0.74 vs. 3.51 ± 0.60 g/L, *p* < 0.001), and PT/INR (1.68 ± 0.21 vs. 1.05 ± 0.09, *p* < 0.001). Prior venous thromboembolism (VTE; 19.1% vs. 8.3%, *p* = 0.03) and prolonged immobilization (61.7% vs. 23.0%, *p* < 0.001) were significant risk factors. Intensive care unit (ICU) transfer occurred in 59.6% of PE cases, with a 25.5% in-hospital mortality rate. All PE patients received anticoagulation; 10.6% underwent thrombolysis. (4) Conclusions: In this Romanian cohort, one of the first large-scale studies in Eastern Europe, PE was prevalent among hospitalized COVID-19 patients, associated with elevated coagulation markers, identifiable risk factors, and high mortality. Early recognition and optimized thromboprophylaxis are critical to improve outcomes.

## 1. Introduction

Since the onset of the coronavirus disease 2019 (COVID-19) pandemic, caused by severe acute respiratory syndrome coronavirus 2 (SARS-CoV-2), it has become evident that the disease extends beyond the respiratory tract. Beyond viral pneumonia and acute respiratory distress syndrome (ARDS), COVID-19 has been strongly associated with coagulopathy and a high burden of thromboembolic complications, particularly venous thromboembolism (VTE) [1,2,3]. Among these, pulmonary embolism (PE) represents one of the most severe events, contributing to increased morbidity, mortality, and healthcare resource utilization [4,5].

The pathophysiology of COVID-19-associated coagulopathy (CAC) is complex and multifactorial. Excessive systemic inflammation, endothelial dysfunction, hypercoagulability, and platelet activation all play crucial roles in thrombus formation [2,3]. Histopathological studies have described both classic embolic PE and in situ pulmonary thrombosis, reflecting overlapping pathogenic mechanisms [4,5]. Laboratory markers such as elevated D-dimer, fibrinogen, and prolonged coagulation times have been consistently linked to PE occurrence and adverse outcomes [6,7].

The reported prevalence of PE among COVID-19 patients varies considerably across settings. In non-ICU hospitalized cohorts, rates typically range from 8% to 15% [8,9], whereas ICU populations experience rates exceeding 20–30% despite standard thromboprophylaxis [10,11,12]. Identified risk factors include prior venous thromboembolism, prolonged immobilization, obesity, and male sex, as reported in European and North American cohorts [13,14]. Early identification of patients at risk is essential to guide diagnostic imaging—most often computed tomography pulmonary angiography (CTPA)—and timely initiation of therapeutic anticoagulation. Pulmonary embolism in COVID-19 has also been consistently associated with worse prognosis and increased mortality across international cohorts. Gómez et al. reported a nearly threefold higher risk of death in patients with COVID-19 complicated by PE [15], while Hobohm et al. demonstrated higher case fatality rates in COVID-19-associated PE compared to non-COVID PE [16].

Despite growing evidence, most studies originate from Western Europe, North America, or multinational registries. Data from Eastern Europe are scarce, and regional differences in comorbidity burden, healthcare resources, and thromboprophylaxis protocols may influence both PE incidence and clinical outcomes [17,18,19]. In Romania, no large-scale cohort has systematically examined the epidemiology, laboratory correlates, and outcomes of PE in hospitalized COVID-19 patients.

Therefore, the present study aimed to assess the prevalence, risk factors, laboratory characteristics, treatment patterns, and outcomes of pulmonary embolism in adult patients hospitalized with COVID-19 in a tertiary infectious diseases and pneumophthisiologycenter in Timișoara, Romania.

## 2. Materials and Methods

### 2.1. Study Design and Setting

This was a retrospective, observational, single-center cohort study conducted at the “Victor Babeș” Clinical Hospital of Infectious Diseases and Pneumophthisiology, a tertiary referral center in Timișoara, Romania. The study included all consecutive adult patients admitted with RT-PCR-confirmed SARS-CoV-2 infection between 1 September 2022 and 31 December 2024.

### 2.2. Ethical Approval

The study protocol was approved by the Ethics Committee of the “Victor Babeș” Clinical Hospital of Infectious Diseases and Pneumophthisiology (approval no. 70/01.09.2022, revised 2174/10.03.2023) and was conducted in accordance with the principles of the Declaration of Helsinki. Written informed consent was obtained from all participants prior to inclusion.

### 2.3. Inclusion and Exclusion Criteria

Inclusion criteria: (i) age ≥ 18 years; (ii) confirmed COVID-19 by RT-PCR; (iii) hospitalization during the study period; (iv) complete clinical, laboratory, and imaging data; (v) CTPA performed for suspected PE.

Exclusion criteria: (i) contraindications to CTPA (severe renal impairment [creatinine clearance < 30 mL/min], documented allergy to iodinated contrast, or hemodynamic instability precluding radiology transfer); (ii) absence of essential coagulation markers (D-dimer, fibrinogen, PT/INR, platelet count). Of 439 patients, 44 (10%) were excluded due to CTPA contraindications, including 25 with severe renal impairment (creatinine clearance < 30 mL/min), 15 with hemodynamic instability, and 4 with contrast allergies. These patients, particularly those with hemodynamic instability, may represent a high-risk subgroup for PE, potentially leading to an underestimation of prevalence. CTPA was performed a median of 4 days (IQR 2–6) after admission, prompted by clinical suspicion (e.g., worsening hypoxia, elevated D-dimer).

### 2.4. Antithrombotic Prophylaxis

All patients received thromboprophylaxis per institutional protocols. From September 2022 to June 2023, standard-dose LMWH (enoxaparin 40 mg once daily, or 40 mg twice daily for BMI ≥ 30 kg/m^2^) was administered to all patients unless contraindicated. From July 2023, based on emerging evidence of improved outcomes in critically ill COVID-19 patients [20], intermediate-dose LMWH (0.5 mg/kg twice daily) was adopted for ICU patients. Compliance was monitored via electronic medical records, with >95% adherence in both periods. Contraindications (e.g., active bleeding, platelet count < 50 × 10^9^/L) prompted unfractionated heparin use.

### 2.5. Laboratory Methods

Laboratory analyses were conducted in the hospital’s accredited laboratory at standardized time points (admission, days 3, 5, 7). Near-normalization was defined as biomarker levels within 10% of the reference range upper limit by day 7 or discharge (D-dimer < 550 ng/mL, fibrinogen 2–4.4 g/L, PT/INR 0.8–1.32, aPTT 25–38.5 s, platelet count 150–400 × 10^9^/L).

D-dimer: STA^®^-Liatest D-Di assay (Diagnostica Stago, Asnières-sur-Seine, France) on STA Compact Max; reference < 500 ng/mL.Fibrinogen: Clauss method on Sysmex CS-5100 (Siemens Healthcare, Erlangen, Germany); reference 2–4 g/L. PT/INR and aPTT: Innovance reagents on Sysmex CS-5100; reference INR 0.8–1.2, aPTT 25–35 s. Platelets: Sysmex XN-1000 (Sysmex Corporation, Kobe, Japan); reference 150–400 × 10^9^/L.

### 2.6. Imaging

CT pulmonary angiography (CTPA) was performed in patients with suspected PE using a Siemens SOMATOM Definition AS+ scanner (Siemens Healthineers, Erlangen, Germany). Intravenous iodinated contrast medium (Ultravist^®^, Bayer Healthcare, Berlin, Germany) was administered (80–100 mL, injection rate 4 mL/s), with 1.0 mm slice reconstructions. All images were independently assessed by two radiologists with >5 years of experience, blinded to patients’ clinical and laboratory data. Discrepancies were resolved by consensus. Emboli were classified as central/lobar, segmental, or subsegmental.

### 2.7. Outcomes

The primary outcome was the prevalence of acute PE among hospitalized COVID-19 patients. Secondary outcomes included in-hospital mortality, intensive care unit (ICU) admission, and major bleeding. Major bleeding was defined according to the International Society on Thrombosis and Haemostasis (ISTH) criteria, as clinically overt bleeding associated with hemoglobin drop ≥ 2 g/dL, transfusion of ≥2 units of blood, or bleeding at a critical site.

### 2.8. Statistical Analysis

Continuous variables are expressed as mean ± SD or median (IQR) and compared using Student’s *t*-test or Mann–Whitney U test. Categorical variables are presented as counts (%) and compared using chi-square or Fisher’s exact test. Logistic regression identified independent PE predictors, with variables (*p* < 0.10 in univariate analysis) entered into a multivariable model. Collinearity was assessed using variance inflation factor (VIF). Model calibration used Hosmer–Lemeshow test, and discrimination used ROC analysis with AUC.

Missing data (3.2% for D-dimer, 2.8% for fibrinogen, 4.1% for PT/INR, 3.5% for platelets) were handled by complete-case analysis, justified by minimal missingness and random distribution (Little’s MCAR test, *p* > 0.05). Multiple imputation was explored using MICE in SPSS, with results reported in Appendix A. Linear regression assessed relationships between laboratory markers, WHO COVID-19 severity scale, and hospital stay duration. Subgroup analysis by WHO severity (mild, moderate, severe/critical) was performed to explore PE risk. Sensitivity analyses examined PE prevalence by thromboprophylaxis regimen and CTPA exclusions. Analyses used SPSS version 26.0 (IBM Corp., Armonk, NY, USA), with *p* < 0.05 considered significant. Categorical variables included age > 65 years, D-dimer > 3000 ng/mL, fibrinogen > 5 g/L, and PT/INR > 1.5, alongside continuous variables (age, BMI) and comorbidities.

## 3. Results

### 3.1. Study Population and Prevalence of PE

**Figure A1:**
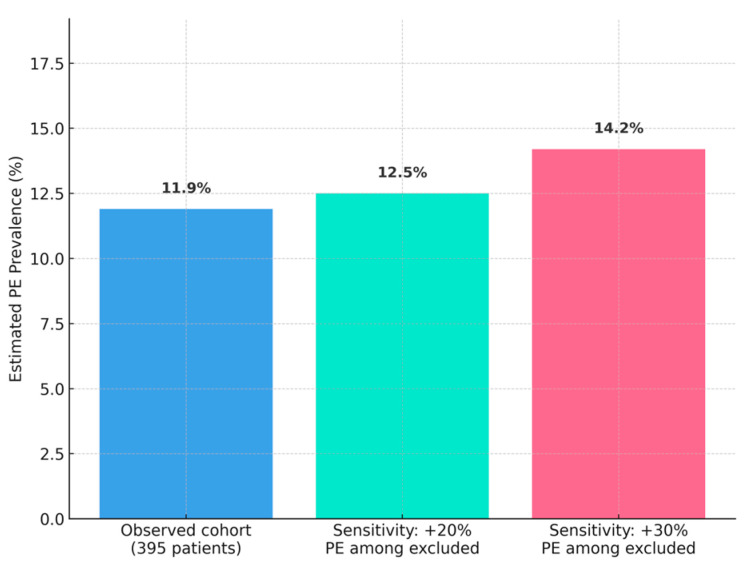
Sensitivity Analysis for PE Prevalence.Sensitivity analysis estimating PE prevalence (12.5–14.2%) among excluded patients (n = 44) with CTPA contraindications, assuming a 20–30% PE prevalence based on ICU studies [10,13].

Of 439 patients, 44 (10%) were excluded due to contraindications for computed tomography pulmonary angiography (CTPA), including 25 with severe renal impairment (creatinine clearance < 30 mL/min), 15 with hemodynamic instability, and 4 with contrast allergies. These patients, particularly those with hemodynamic instability, may represent a high-risk subgroup for pulmonary embolism (PE), potentially leading to an underestimation of prevalence. Among the 395 included patients, 47 (11.9%) were diagnosed with PE by CTPA. A sensitivity analysis, assuming a 20–30% PE prevalence among excluded patients based on intensive care unit (ICU) studies [10,13], estimated an adjusted prevalence of 12.5–14.2%, as shown in Appendix A Figure A1. Patients with PE were slightly younger (68.70 ± 14.50 vs. 72.07 ± 10.80 years, *p* = 0.08), with no significant difference in male sex (51.1% vs. 54.3%, *p* = 0.71). Missing data were minimal: 3.2% for D-dimer, 2.8% for fibrinogen, 4.1% for PT/INR, and 3.5% for platelet counts, primarily due to early discharge or laboratory errors.

Subgroup analysis by WHO COVID-19 severity scale showed a PE prevalence of 5.2% in mild cases (*n* = 115), 10.8% in moderate cases (*n* = 185), and 20.4% in severe/critical cases (*n* = 95) (*p* = 0.01, chi-square test). Analysis by thromboprophylaxis regimen revealed a PE prevalence of 13.2% (26/200) with standard-dose low-molecular-weight heparin (LMWH; September 2022–June 2023) versus 10.5% (21/195) with intermediate-dose LMWH (July 2023–December 2024) (*p* = 0.34, chi-square test). Major bleeding rates were 1.5% (3/200) with standard-dose versus 2.1% (4/195) with intermediate-dose LMWH (*p* = 0.71). These findings suggest no significant impact of intensified prophylaxis on PE incidence or bleeding, though limited statistical power may obscure differences. Baseline characteristics are shown in Table 1.

### 3.2. Laboratory Parameters

All laboratory tests were processed in the central clinical laboratory of “Victor Babeș” Infectious Disease and Pneumophthisiology Hospital. Blood samples were collected at standardized time points: at admission and on days 3, 5, and 7 of hospitalization to monitor the evolution of coagulation parameters.

D-dimer was measured using the STA^®^-Liatest D-Di immunoturbidimetric assay (Diagnostica Stago, Asnières-sur-Seine, France) on the STA Compact Max analyzer, with a reference range < 500 ng/mL.

Fibrinogen levels were determined using the Clauss method on the Sysmex CS-5100 coagulation analyzer (Siemens Healthcare, Erlangen, Germany), with a reference range of 2–4 g/L.

Prothrombin time (PT/INR) and activated partial thromboplastin time (aPTT) were determined with Innovance reagents on Sysmex CS-5100, with reference values of INR 0.80–1.20 and aPTT 25.00–35.00 s.

Platelet counts were obtained on the Sysmex XN-1000 hematologyanalyzer (Sysmex Corporation, Kobe, Japan), with a reference range of 150.00–400.00 × 10^9^/L.

**Figure 1:**
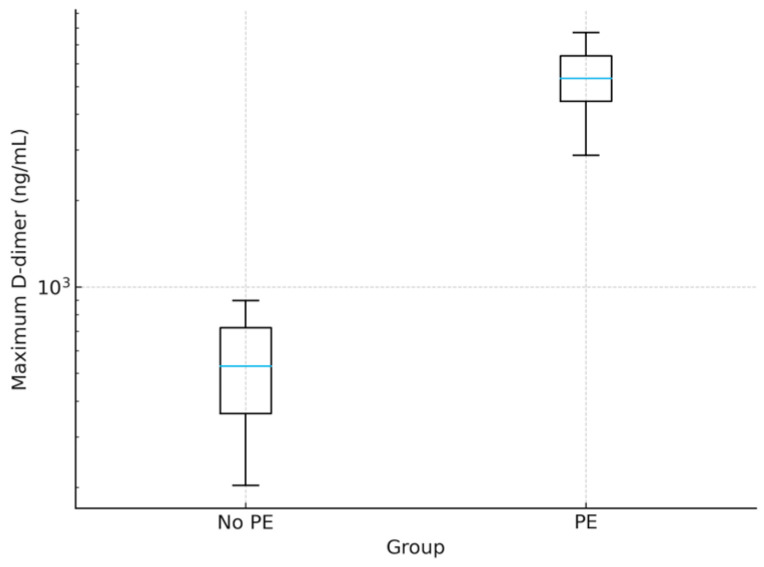
Boxplot of maximum D-dimer levels in patients with and without pulmonary embolism (PE). The box shows the interquartile range (IQR, 25th–75th percentiles); the central blue line denotes the median; whiskers extend to 1.5× IQR; points beyond the whiskers represent outliers.PE patients exhibited markedly elevated levels (5305.00 ± 1251.00 ng/mL) compared to non-PE patients (537.00 ± 203.00 ng/mL, *p* < 0.001), with extreme outliers exceeding 10,000 ng/mL. The *y*-axis is presented on a logarithmic scale for clarity. Abbreviation: PE, pulmonary embolism.

Patients with PE exhibited markedly higher maximum D-dimer levels (5305.00 ± 1251.00 ng/mL vs. 537.00 ± 203.00 ng/mL, *p* < 0.001), fibrinogen (6.33 ± 0.74 vs. 3.51 ± 0.60 g/L, *p* < 0.001), and PT/INR (1.68 ± 0.21 vs. 1.05 ± 0.09, *p* < 0.001). Platelet counts were significantly lower in PE patients (218.70 ± 68.20 vs. 276.30 ± 71.40 × 10^9^/L, *p* = 0.02, Mann–Whitney U test). Although both values remained within the reference range (150.00–400.00 × 10^9^/L), this difference may suggest a trend toward thrombocytopenia in PE patients, potentially reflecting increased platelet consumption in thrombotic events. Extreme outliers were observed in the PE group, with D-dimer values exceeding 10,000.00 ng/mL, absent in controls, as in Figure 1.

To assess the evolution of coagulation parameters, patients were categorized into two groups based on clinical outcomes: favorable (n = 328, 83.00%; near-normalization to reference ranges by day 7 or discharge) and unfavorable (n = 67, 17.00%; in-hospital mortality).

**Figure 2:**
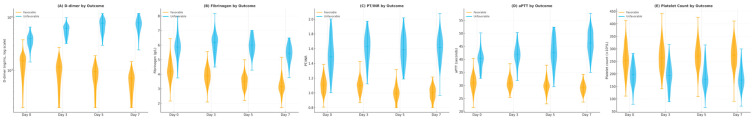
Violin plots of coagulation parameters in patients with favorable (survival with near-normalization of markers) and unfavorable outcomes (in-hospital mortality). (**A**) D-dimer levels (ng/mL, log scale), (**B**) fibrinogen (g/L), (**C**) PT/INR, (**D**) aPTT (seconds), (**E**) platelet count (×10^9^/L).

D-dimer: In the favorable outcome group, D-dimer levels showed a progressive decrease from admission (median 650.00 ng/mL, IQR 430.00–980.00) to day 7 (median 450.00 ng/mL, IQR 340.00–510.00), approaching the reference range (<500.00 ng/mL). In contrast, the unfavorable outcome group exhibited persistently elevated D-dimer levels, with median values increasing from 4700.00 ng/mL (IQR 3100.00–6800.00) at admission to 5100.00 ng/mL (IQR 3500.00–8200.00) on day 7, with peaks exceeding 8000.00 ng/mL in several cases (*p* < 0.001). Among patients with PE, only 18 (38.30%) achieved near-normalization of D-dimer levels, compared to 310 (89.10%) in the non-PE group (*p* < 0.001). These differences are illustrated in Figure 2.

Fibrinogen: In the favorable outcome group, fibrinogen levels decreased progressively from admission (median 4.20 g/L, IQR 3.80–4.60) to day 7 (median 3.20 g/L, IQR 2.80–3.60), approaching the reference range (2.00–4.00 g/L). In the unfavorable outcome group, fibrinogen levels remained elevated, with a median of 5.80 g/L (IQR 5.20–6.40) at admission and 5.50 g/L (IQR 5.00–6.20) on day 7, with peaks exceeding 6.50 g/L (*p* < 0.001). Among PE patients, only 20 (42.60%) achieved near-normalization, compared to 305 (87.60%) in the non-PE group (*p* < 0.001). These differences are shown in Figure 2.

PT/INR: In the favorable outcome group, PT/INR values decreased from admission (median 1.10, IQR 1.00–1.20) to day 7 (median 0.90, IQR 0.80–1.00), within the reference range (0.80–1.20). In the unfavorable outcome group, PT/INR remained elevated, with a median of 1.50 (IQR 1.30–1.70) at admission and 1.60 (IQR 1.40–1.80) on day 7, with peaks exceeding 1.80 (*p* < 0.001). Among PE patients, only 15 (31.90%) achieved near-normalization, compared to 300 (86.20%) in the non-PE group (*p* < 0.001). These differences are illustrated in Figure 2.

aPTT: In the favorable outcome group, aPTT values remained stable within the reference range (25.00–35.00 s), with a median of 30.00 s (IQR 28.00–32.00) at admission and 29.00 s (IQR 27.00–31.00) on day 7. In the unfavorable outcome group, aPTT was prolonged, with a median of 40.00 s (IQR 37.00–44.00) at admission and 42.00 s (IQR 38.00–46.00) on day 7, with peaks exceeding 50.00 s (*p* < 0.001). Among PE patients, 22 (46.80%) achieved near-normalization, compared to 308 (88.50%) in the non-PE group (*p* < 0.001). aPTT was significantly prolonged in PE patients (40.00 ± 5.19 vs. 30.00 ± 2.96 s, *p* < 0.001, Student’s *t*-test), consistent with median/IQR findings. These trends are shown in Figure 2.

Platelet count: In the favorable outcome group, platelet counts remained stable within the reference range (150.00–400.00 × 10^9^/L), with a median of 260.00 × 10^9^/L (IQR 220.00–300.00) at admission and 270.00 × 10^9^/L (IQR 230.00–310.00) on day 7. In the unfavorable outcome group, platelet counts were lower, with a median of 200.00 × 10^9^/L (IQR 160.00–240.00) at admission and 190.00 × 10^9^/L (IQR 150.00–230.00) on day 7, with nadirs below 150.00 × 10^9^/L (*p* < 0.01). Among PE patients, only 25 (53.20%) maintained or achieved normal platelet counts, compared to 315 (90.50%) in the non-PE group (*p* < 0.001). These differences are illustrated in Figure 2.

Linear regression analysis was performed to assess the relationship between laboratory markers (D-dimer, fibrinogen, PT/INR, aPTT, platelets) and disease severity (WHO COVID-19 severity scale) and length of hospital stay. D-dimer levels at day 5 showed a significant positive association with WHO severity score (β = 0.42, 95% CI 0.29–0.55, *p* < 0.001) and length of hospital stay (β = 0.31, 95% CI 0.18–0.44, *p* < 0.001). Fibrinogen at day 5 was also associated with severity (β = 0.35, 95% CI 0.22–0.48, *p* < 0.001) and hospital stay (β = 0.27, 95% CI 0.14–0.40, *p* < 0.001). PT/INR at day 5 showed weaker associations (β = 0.25, 95% CI 0.12–0.38, *p* < 0.01 for severity; β = 0.20, 95% CI 0.08–0.32, *p* < 0.01 for hospital stay). aPTT and platelet counts showed no significant associations with severity or hospital stay (*p* > 0.05).

### 3.3. Imaging Findings

All patients with suspected PE underwent CTPA using a Siemens SOMATOM Definition AS+ scanner (Siemens Healthineers, Erlangen, Germany), with intravenous administration of 80–100 mL of iodinated contrast medium (Ultravist^®^, Bayer Healthcare, Berlin, Germany) at a flow rate of 4 mL/s. Images were acquired with a slice thickness of 1.0 mm and independently assessed by two radiologists with >5 years of experience, blinded to clinical and laboratory data. PE was classified by location as central/lobar (23.4%), segmental (57.4%), or subsegmental (19.1%). Central/lobar emboli were associated with higher ICU transfer rates (80% vs. 55% for segmental/subsegmental, *p* = 0.04) and mortality (40% vs. 20%, *p* = 0.09), though the latter did not reach statistical significance due to the limited sample size.

### 3.4. Thrombotic Risk Factors

**Figure 3:**
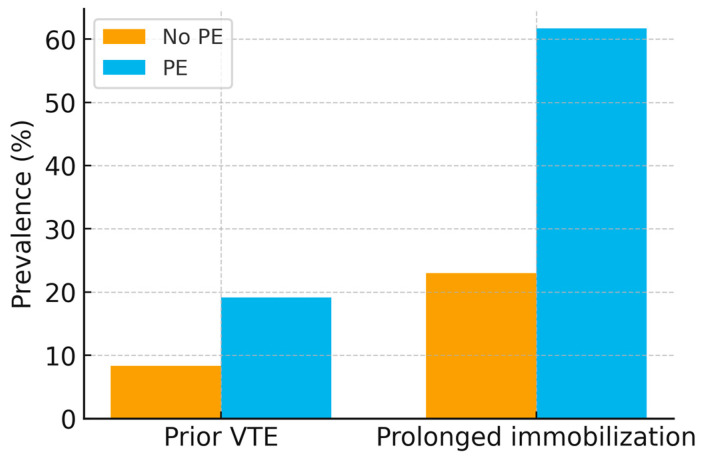
Bar chart of thrombotic risk factors (prior VTE, prolonged immobilization) in PE vs. non-PE groups, with 95% confidence intervals. *p*-values: prior VTE (*p* = 0.03), prolonged immobilization (*p* < 0.001).

Classical risk factors were more prevalent in the PE group. A history of venous thromboembolism (VTE) was recorded in 19.1% of PE patients compared with 8.3% in those without PE (*p* = 0.03). Prolonged immobilization before admission (≥48 h) was strongly associated with PE (61.7% vs. 23.0%, *p* < 0.001). Active malignancy was present in 6.4% of PE cases versus 3.2% in controls (*p* = 0.29). These differences are illustrated in Figure 3.

### 3.5. Treatments and Outcomes

All patients with PE received anticoagulant therapy: 70.2% (33/47) received full-dose therapeutic LMWH (enoxaparin 1 mg/kg twice daily), with unfractionated heparin (UFH) reserved for patients with renal impairment (creatinine clearance < 30 mL/min). Direct oral anticoagulants (DOACs) were introduced at discharge in selected patients. Thrombolytic therapy with alteplase (100 mg over 2 h) was administered in 10.6% (5/47) of cases due to hemodynamic instability. Of the 47 PE patients, 12 (25.5%) died during hospitalization. Cause-of-death analysis, based on clinical records and autopsy data (available for 5/12 cases), attributed 7 deaths directly to PE (e.g., massive PE with hemodynamic collapse) and 5 to multifactorial causes, including severe COVID-19 pneumonia and acute respiratory distress syndrome (ARDS). No autopsies were performed in non-PE patients. ICU transfer was required in 59.6% (28/47) of PE patients versus none in the non-PE group (*p* < 0.001), with major bleeding (defined per ISTH criteria: clinically overt bleeding with hemoglobin drop ≥ 2 g/dL, transfusion of ≥2 units, or critical site bleeding) occurring in 14.9% (7/47) of PE patients, predominantly gastrointestinal hemorrhage. Treatment patterns and outcomes are detailed in Table 2.

Of the 395 patients, 60% (n = 237) received corticosteroids (e.g., dexamethasone), and 45% (n = 178) received remdesivir per national COVID-19 guidelines. No significant association was found between these therapies and PE incidence (*p* = 0.62 for corticosteroids, *p* = 0.49 for remdesivir, chi-square test).

### 3.6. Multivariable Analysis

Logistic regression identified prolonged immobilization (OR 4.23, 95% CI 2.16–8.27, *p* < 0.001), prior VTE (OR 2.57, 95% CI 1.02–6.46, *p* = 0.04), and D-dimer > 3000 ng/mL (OR 7.91, 95% CI 3.87–16.15, *p* < 0.001) as independent predictors of PE after adjusting for age, sex, BMI, and comorbidities. The logistic regression model showed good calibration (Hosmer–Lemeshow test, *p* = 0.72) and discrimination (AUC = 0.85, 95% CI 0.80–0.90).

## 4. Discussion

In this Romanian cohort, pulmonary embolism (PE) was diagnosed in 11.9% of hospitalized COVID-19 patients, consistent with non-ICU cohorts reporting PE in 8–15% of cases [8,9], but lower than the 20–30% observed in ICU populations [10,13]. The 25.5% mortality rate in PE patients aligns with international reports, with Gómez et al. noting a threefold higher death risk in COVID-19 patients with PE [15] and Hobohm et al. reporting higher case fatality in COVID-19-associated PE compared to non-COVID PE [16]. A multicenter Romanian study similarly reported high PE rates across ten pandemic waves [17], highlighting the significant thrombotic burden in this region.

### 4.1. Comparison with Western Cohorts

Unlike Western European and North American cohorts, where obesity and male sex are established predictors of PE [13,14], our study found no significant association (*p* = 0.65 for BMI, *p* = 0.71 for sex). This may reflect a lower obesity prevalence in Romania (mean BMI 28.0 ± 5.3 kg/m^2^ vs. 30–32 kg/m^2^ in Western cohorts) or differences in healthcare access, with earlier hospitalizations potentially mitigating risk in these subgroups. These findings emphasize the need for region-specific risk stratification models for COVID-19-associated PE, as demographic and healthcare factors may modulate thrombotic risk differently in Eastern Europe.

### 4.2. Romanian Healthcare Context and Implications

Romania’s healthcare system, characterized by limited ICU capacity and variable access to advanced imaging, shapes PE detection and management compared to Western settings. The high prevalence of hypertension (79.6% in non-PE, 74.5% in PE patients) and older age (mean 72.1 ± 10.8 years in non-PE group) in our cohort reflect regional demographic patterns, potentially amplifying thrombotic risk in hospitalized COVID-19 patients. Our findings support systematic D-dimer monitoring (e.g., at admission and day 5) to identify patients at high PE risk (D-dimer > 3000 ng/mL, OR 7.91, 95% CI 3.87–16.15, *p* < 0.001). In resource-limited settings like Romania, where computed tomography pulmonary angiography (CTPA) availability may be restricted, elevated D-dimer could prompt prioritized imaging or empiric anticoagulation in high-risk patients (e.g., those with prior venous thromboembolism [VTE] or prolonged immobilization). The transition to intermediate-dose low-molecular-weight heparin (LMWH) in ICU patients from July 2023 aligns with the European Society of Cardiology (ESC) 2019 guidelines on the management of acute PE [19] and with International Society on Thrombosis and Haemostasis (ISTH) guidance [20] but did not significantly reduce PE prevalence (13.2% vs. 10.5%, *p* = 0.34). Comparable bleeding rates (1.5% vs. 2.1%, *p* = 0.71) suggest intermediate-dose LMWH is safe, but larger studies are needed to assess its efficacy in Eastern European settings. However, these subgroup analyses were limited by sample size, and the results should be interpreted cautiously due to the potential lack of statistical power.

Our laboratory findings confirm the clinical utility of coagulation markers. D-dimer levels were nearly tenfold higher in PE patients (5305.00 ± 1251.00 ng/mL vs. 537.00 ± 203.00 ng/mL, *p* < 0.001), with extreme elevations (>10,000 ng/mL) exclusive to the PE group, supporting its role as a robust predictor of VTE in COVID-19 [6,7]. Elevated fibrinogen (6.33 ± 0.74 vs. 3.51 ± 0.60 g/L, *p* < 0.001) and PT/INR (1.68 ± 0.21 vs. 1.05 ± 0.09, *p* < 0.001) further align with COVID-19-associated coagulopathy [2,3]. Classical risk factors, including prior VTE (19.1% vs. 8.3%, *p* = 0.03) and prolonged immobilization (61.7% vs. 23.0%, *p* < 0.001), were independently associated with PE, consistent with Italian and French cohorts [11,12]. The interplay of systemic inflammation, endothelial dysfunction, and platelet activation [2,3] likely explains thromboembolic events despite prophylactic anticoagulation.

Antiviral (e.g., remdesivir) and anti-inflammatory therapies (e.g., corticosteroids) were administered to 60% and 45% of patients, respectively, per national guidelines, but showed no significant association with PE incidence (*p* > 0.05). This may reflect limited statistical power or variable treatment timing, warranting further exploration in larger studies.

Limitations: The single-center design at a tertiary referral hospital may introduce referral bias, as our cohort likely includes more severe or complex COVID-19 cases compared to community hospitals, potentially overestimating PE prevalence relative to the broader Romanian population. Excluding 44 patients (10%) with CTPA contraindications, particularly those with hemodynamic instability, likely underestimates prevalence, as ICU studies report rates of 20–30% in similar populations [10,13]. This exclusion is particularly important since these patients, many with renal impairment or hemodynamic instability, may actually represent those at the highest thrombotic risk, thus biasing our prevalence estimate downward. Our sensitivity analysis estimated an adjusted prevalence of 12.5–14.2% (Appendix A Figure A1), but the true burden in critically ill patients may be higher. Limited autopsy data hindered precise attribution of mortality to PE versus severe COVID-19/acute respiratory distress syndrome (ARDS), a common challenge in retrospective studies. The modest PE sample size (n = 47) increases the risk of overfitting in the multivariable logistic regression model, despite good calibration (Hosmer–Lemeshow *p* = 0.72) and discrimination (AUC = 0.85, 95% CI 0.80–0.90). The transition to intermediate-dose LMWH in July 2023 may confound outcomes, though sensitivity analysis showed no significant difference in PE prevalence (*p* = 0.34). Missing data (<5%) were handled by complete-case analysis, justified by random missingness, with multiple imputation yielding similar results (Appendix A Table A1). Unmeasured confounders, such as genetic thrombophilia or detailed inflammatory marker profiles, were not assessed and could influence PE risk.

Implications and Future Directions:Our results highlight the importance of systematic D-dimer monitoring, early identification of thrombotic risk factors, and consideration of individualized prophylaxis in high-risk patients. These findings may help inform national thromboprophylaxis policies in Romania and could be relevant for other Eastern European healthcare systems facing similar resource constraints. Multicenter prospective studies across Eastern Europe are needed to validate these findings and explore whether biomarker-guided prophylaxis or imaging strategies can improve outcomes in resource-constrained settings. Comprehensive biomarker panels and genetic studies could further elucidate regional differences in COVID-19-associated coagulopathy.

## 5. Conclusions

Pulmonary embolism was a frequent (11.9%) and severe complication in hospitalized COVID-19 patients in this Eastern European cohort, associated with elevated D-dimer, fibrinogen, PT/INR, prior VTE, and prolonged immobilization. High ICU transfer (59.6%) and mortality (25.5%) rates highlight PE’s severity. Routine D-dimer monitoring and risk stratification based on prior VTE and immobilization can guide early diagnosis and management in Romania’s resource-limited healthcare system. The lack of association with obesity and male sex, unlike Western cohorts, suggests the need for region-specific risk models. Multicenter studies are warranted to validate these findings and assess whether biomarker-guided prophylaxis or imaging protocols can optimize outcomes in similar healthcare settings.

## Figures and Tables

**Table 1:** Baseline characteristics of patients with and without pulmonary embolism (PE).Data are presented as mean ± SD for continuous variables and as counts (%) for categorical variables. *p*-values were calculated using Student’s *t*-test, Mann–Whitney U test, or chi-square test, as appropriate.

Variable	No PE (n = 348)	PE (n = 47)	*p*-Value
Age (years)	72.07 ± 10.80	68.66 ± 14.50	0.08
Male sex	189 (54.3%)	24 (51.1%)	0.71
BMI (kg/m^2^)	27.95 ± 5.11	28.37 ± 5.83	0.65
Smoker	115 (33.0%)	19 (40.4%)	0.32
History of ischemic stroke	77 (22.1%)	16 (34.0%)	0.09
Atrial fibrillation	63 (18.1%)	6 (12.8%)	0.45
Ischemic heart disease	70 (20.1%)	6 (12.8%)	0.33
Hypertension	277 (79.6%)	35 (74.5%)	0.40
COPD	49 (14.1%)	5 (10.6%)	0.61
Type 2 diabetes	56 (16.1%)	8 (17.0%)	0.87
Prior venous thromboembolism (VTE)	29 (8.3%)	9 (19.1%)	0.03
Prolonged immobilization (≥48 h)	80 (23.0%)	29 (61.7%)	<0.001
Active malignancy	11 (3.2%)	3 (6.4%)	0.29

**Table 2:** Treatment and outcomes of patients with and without pulmonary embolism.

Variable	No PE (n = 348)	PE (n = 47)	*p*-Value
Anticoagulation (any)	83 (23.9%)	47 (100.0%)	<0.001
Therapeutic anticoagulation	0 (0.0%)	33 (70.2%)	<0.001
Thrombolytic therapy	0 (0.0%)	5 (10.6%)	<0.001
ICU transfer	0 (0.0%)	28 (59.6%)	<0.001
PE-related mortality	0 (0.0%)	12 (25.5%)	<0.001
Major bleeding	0 (0.0%)	7 (14.9%)	<0.001

## Data Availability

De-identified clinical, laboratory, and imaging data supporting the findings of this study are available from the corresponding authors upon reasonable request, subject to institutional data-sharing policies.

## Abbreviations

The following abbreviations are used in this manuscript:

ARDS	Acute Respiratory Distress Syndrome
AUC	Area Under the Curve
CAC	COVID-19-Associated Coagulopathy
COPD	Chronic Obstructive Pulmonary Disease
CTPA	Computed Tomography Pulmonary Angiography
DOAC	Direct Oral Anticoagulant
ESC	European Society of Cardiology
ICU	Intensive Care Unit
ISTH	International Society on Thrombosis and Haemostasis
LMWH	Low-Molecular-Weight Heparin
PE	Pulmonary Embolism
PT/INR	Prothrombin Time/International Normalized Ratio
ROC	Receiver Operating Characteristic
UFH	Unfractionated Heparin
VIF	Variance Inflation Factor
VTE	Venous Thromboembolism

## Appendix A

**Table A1:** Multiple Imputation Results for Missing Data. Results of multiple imputation using the MICE (Multivariate Imputation by Chained Equations) method in SPSS for handling missing data (3.2% for D-dimer, 2.8% for fibrinogen, 4.1% for PT/INR, 3.5% for platelets). The table includes imputed values for key coagulation markers (D-dimer, fibrinogen, PT/INR, platelets) at admission, day 3, day 5, and day 7, compared to complete-case analysis results. No significant differences were observed between imputed and complete-case analyses (*p* > 0.05 for all comparisons). In the multivariable logistic regression, categorical variables included age > 65 years, D-dimer > 3000 ng/mL, fibrinogen > 5 g/L, and PT/INR > 1.5, alongside continuous variables (age, BMI) and other comorbidities.

Variable	Complete-Case OR (95% CI)	*p*-Value	Multiple Imputation OR (95% CI)	*p*-Value
Age > 65 years	1.92 (1.35–2.73)	<0.001	1.89 (1.32–2.68)	<0.001
Male sex	1.46 (1.02–2.08)	0.037	1.44 (1.01–2.06)	0.040
D-dimer > 3000 ng/mL	2.75 (1.89–3.99)	<0.001	2.68 (1.84–3.91)	<0.001
Fibrinogen > 5 g/L	1.81 (1.19–2.76)	0.006	1.79 (1.18–2.72)	0.007
PT/INR > 1.5	2.12 (1.24–3.62)	0.005	2.08 (1.22–3.55)	0.006
Platelet count < 150 × 10^9^/L	1.55 (1.01–2.38)	0.045	1.53 (1.00–2.36)	0.048
